# Reconstitution reveals Ykt6 as the autophagosomal SNARE in autophagosome–vacuole fusion

**DOI:** 10.1083/jcb.201804028

**Published:** 2018-10-01

**Authors:** Levent Bas, Daniel Papinski, Mariya Licheva, Raffaela Torggler, Sabrina Rohringer, Martina Schuschnig, Claudine Kraft

**Affiliations:** 1 Max F. Perutz Laboratories, Vienna Biocenter, University of Vienna, Vienna, Austria; 2 Institute of Biochemistry and Molecular Biology, Centre for Biochemistry and Molecular Cell Research , Faculty of Medicine, University of Freiburg, Freiburg, Germany; 3 Faculty of Biology, University of Freiburg, Freiburg, Germany

## Abstract

Autophagy mediates the bulk degradation of cytoplasmic material, particularly during starvation. Upon the induction of autophagy, autophagosomes form a sealed membrane around cargo, fuse with a lytic compartment, and release the cargo for degradation. The mechanism of autophagosome–vacuole fusion is poorly understood, although factors that mediate other cellular fusion events have been implicated. In this study, we developed an in vitro reconstitution assay that enables systematic discovery and dissection of the players involved in autophagosome–vacuole fusion. We found that this process requires the Atg14–Vps34 complex to generate PI3P and thus recruit the Ypt7 module to autophagosomes. The HOPS-tethering complex, recruited by Ypt7, is required to prepare SNARE proteins for fusion. Furthermore, we discovered that fusion requires the R-SNARE Ykt6 on the autophagosome, together with the Q-SNAREs Vam3, Vam7, and Vti1 on the vacuole. These findings shed new light on the mechanism of autophagosome–vacuole fusion and reveal that the R-SNARE Ykt6 is required for this process.

## Abstract (precis)

Autophagosome fusion with vacuoles requires a conserved fusion machinery, though the topology remained unclear. Two papers in this issue, Bas et al. and Gao et al., uncover Ykt6 as the required autophagosomal SNARE.

## Introduction

Macroautophagy, hereafter referred to as autophagy, is an intracellular degradation and recycling pathway that is highly conserved among eukaryotes. During autophagy, cellular material is engulfed within a newly formed double membrane vesicle, the autophagosome. Once complete, the outer autophagosomal membrane fuses with a lytic compartment, that is, the lysosome in mammals or the vacuole in yeast and plants. Fusion releases the inner vesicle, the so-called autophagic body, for degradation in the lumen of the lytic compartment.

Over 40 autophagy-related (Atg) proteins are known to act in the yeast autophagy pathway, and many functional homologues of these players have been identified in higher eukaryotes (Nakatogawa et al., 2009; Yang and Klionsky, 2010). The core Atg proteins, required for most types of autophagy, are known to regulate all steps of the pathway up to autophagosome maturation. Mature autophagosomes must become competent for fusion, but how this happens and how fusion is achieved at the molecular level remain unknown. Similar to endosome–vacuole fusion, the fusion of autophagosomes with vacuoles requires the action of the RAB7-like GTPase Ypt7, the homotypic vacuole fusion and protein sorting (HOPS) tethering complex, and SNARE proteins (Reggiori and Ungermann, 2017). Ypt7 requires the Mon1–Ccz1 guanine nucleotide exchange factor complex for activation and for stable localization at the vacuole and the autophagosome. Recently, it was reported that Mon1–Ccz1 localizes to autophagosomes with RAB7/Ypt7 in both *Drosophila melanogaster* and yeast (Hegedűs et al., 2016; Gao et al., 2018a). Activated Ypt7 in its GTP-bound form recruits the HOPS complex (Seals et al., 2000), which in turn supports SNARE assembly and fusion (Krämer and Ungermann, 2011). SNARE proteins, Rab7, and the HOPS complex have also been implicated in mammalian autophagy (Shen and Mizushima, 2014). Whether fusion requires Ypt7 at autophagic membranes, the mature autophagosome, the vacuole, or both organelles is unclear.

SNAREs are membrane proteins that have canonical heptad repeat domains (SNARE motifs) with a central argininyl (R) or glutaminyl (Q) residue and therefore are termed R- or Q-SNAREs, respectively. SNARE complexes are composed of four-helix bundles consisting of three Q-SNAREs and one R-SNARE (Fasshauer et al., 1998; Wickner and Rizo, 2017). SNAREs associate on opposing membranes, zipper their SNARE domains, and thereby deform the bilayers, driving lipid rearrangements that lead to membrane fusion. Sec17/α-SNAP and the ATPase Sec18/NSF then release the SNARE bundle and prepare SNARE proteins for the next round of fusion (Wickner and Rizo, 2017). Yeasts with mutations in the Q-SNAREs Vam3, Vam7, and Vti1 have been reported to accumulate mature autophagosomes, suggesting that they might support autophagosome–vacuole fusion (Darsow et al., 1997; Sato et al., 1998; Ishihara et al., 2001). Similarly, these SNAREs seem to be required for late steps in mammalian autophagy (Shen and Mizushima, 2014). Vam7 has been reported to interact with the Atg17–Atg31–Atg29 complex (Liu and Klionsky, 2016). Whether Vam3, Vam7, and Vti1 act on the autophagosome or on the vacuole during autophagosome–vacuole fusion remains to be shown.

SNARE proteins also act in earlier steps in autophagy such as autophagosome formation and closure. The SNAREs required for yeast autophagosome formation are Sso1, Sso2, Sec9, Tlg2, Sec22, and Ykt6 (Abeliovich et al., 1999; Reggiori and Klionsky, 2006; Nair and Klionsky, 2011). Mutants of these SNAREs fail to generate autophagosomes, so whether they are also required for autophagosome–vacuole fusion is unknown. Indeed, it is unclear which SNARE on the yeast autophagosome supports fusion with the vacuole.

Technical limitations have obscured insight into the mechanism of autophagosome–vacuole fusion. For instance, disrupting membrane fusion factors in intact cells may not only affect autophagy directly but will affect vesicular trafficking in general, with potential indirect effects on autophagy. Also, if a fusion factor is required in early steps of autophagosome formation, this requirement will mask its roles in late stages of the pathway such as fusion to the vacuole. As a result, the factors that are required for autophagosome-to-vacuole fusion, where they localize and act, and how they are recruited have remained a mystery.

We have overcome this critical barrier by developing a new approach that recapitulates autophagosome–vacuole fusion in vitro using isolated yeast vacuoles, an autophagosome-enriched fraction, and a cytosolic extract. This system allows us to uncover the players in autophagosome–vacuole fusion and to dissect the molecular steps of autophagosome–vacuole fusion by determining whether a player acts on the autophagosome, on the vacuole, or in the cytosol.

## Results

### Reconstitution of autophagosome–vacuole fusion in vitro

**Figure 1.**
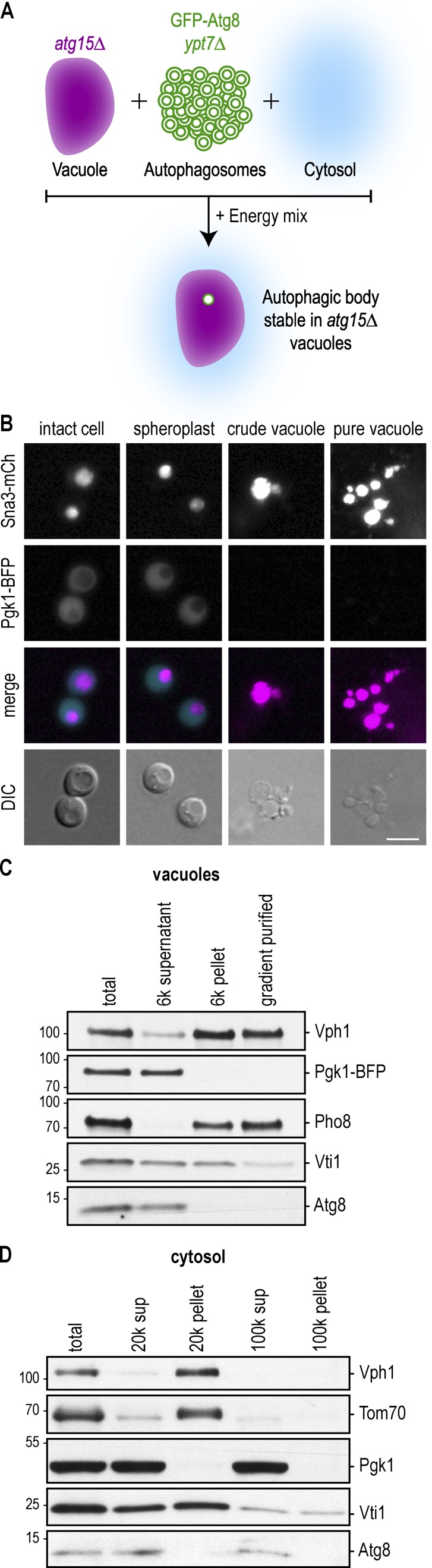
**Preparation of cytosolic and vacuolar fractions. (A)** Schematic of the experimental setup: crude vacuolar, autophagosomal, and cytosolic fractions are individually prepared from yeast cells. Incubation of these three fractions together with an energy regeneration system allows the fusion of autophagosomes with vacuoles in vitro. **(B)** Vacuoles were isolated from Sna3-mCherry Pgk1-BFP *atg15Δ* cells. Logarithmically growing cells were harvested and lysed, and vacuoles were separated from the cytosolic fraction by a 6,000 *g* spin. The pellet containing the vacuoles was then further separated on a 0–4–8–15% Ficoll step gradient, and vacuoles were collected at the 0–4% Ficoll interface. The purification steps were analyzed by fluorescence microscopy. DIC, differential interference contrast. Bar, 5 µm. **(C)** The individual fractions from B were analyzed by anti-Vph1, anti-Pgk1, anti-Pho8, anti-Vti1, and anti-Atg8 Western blotting. One representative experiment out of three is shown. **(D)** Cytosol was prepared from WT cells treated with 220 nM rapamycin for 4 h by freezer milling and centrifugation at 20,000 *g*. The supernatant was further spun at 100,000 *g*. Fractions were analyzed by anti-Vph1, anti-Tom70, anti-Pgk1, anti-Vti1, and anti-Atg8 Western blotting. One representative experiment out of three is shown. Molecular masses are given in kilodaltons.

To discover factors that are involved in autophagosome–vacuole fusion and discern where they function, we aimed to reconstitute this process in vitro. First, we prepared separate fractions containing autophagosomes, vacuoles, and cytosol from *Saccharomyces cerevisiae* to test in an in vitro fusion assay (Fig. 1 A). Of note, we enriched for autophagosomes or vacuoles within crude mixtures, reasoning that these partially purified mixtures would contain additional factors that are potentially required for fusion.

To isolate vacuoles, we enzymatically removed the yeast cell wall, lysed the cells, and collected vacuoles via 6,000 *g* centrifugation (crude vacuoles). We further separated the pellet on a 0–4–8–15% Ficoll step gradient, isolating vacuoles at the 0–4% Ficoll gradient interface as previously described (pure vacuoles; Cabrera and Ungermann, 2008). We used a strain that expresses a Pgk1-BFP fusion protein (cytosolic marker) and a Sna3-mCherry fusion protein (vacuolar marker; Fig. 1 B), which allowed us to visualize successful cell lysis and isolation of vacuoles. In addition, Western blot analysis of the different fractions revealed that the vacuolar markers Vph1, Pho8, and Vti1 were enriched relative to the cytosolic marker Pgk1-BFP in both the crude vacuole pellet and the gradient-purified vacuoles, whereas Pgk1-BFP was mostly found in the supernatant. Vacuoles were isolated from logarithmically growing cells. In nutrient-rich conditions, bulk autophagy is repressed, and thus the vacuolar fractions do not contain bulk autophagosomes. Indeed, the autophagosomal marker Atg8 was found in the supernatant, likely representing unconjugated Atg8 or its conjugation to immature membranes (Fig. 1 C).

To generate a cytosolic extract, we treated WT yeast cells with rapamycin for 4 h and lysed them by freezer milling (Kraft et al., 2012). Rapamycin inhibits Target of rapamycin complex 1, which mimics starvation and induces bulk autophagy (Noda and Ohsumi, 1998). The crude cell extract was cleared by subsequent centrifugations of 20,000 *g* and 100,000 *g* (Fig. 1 D). We detected the cytosolic marker Pgk1 mainly in the supernatant fractions, whereas membrane markers such as Vph1 and Vti1 (vacuoles) and Tom70 (mitochondria) were enriched in the pellet, indicating that the supernatant fraction was largely cleared from membranes.

**Figure 2.**
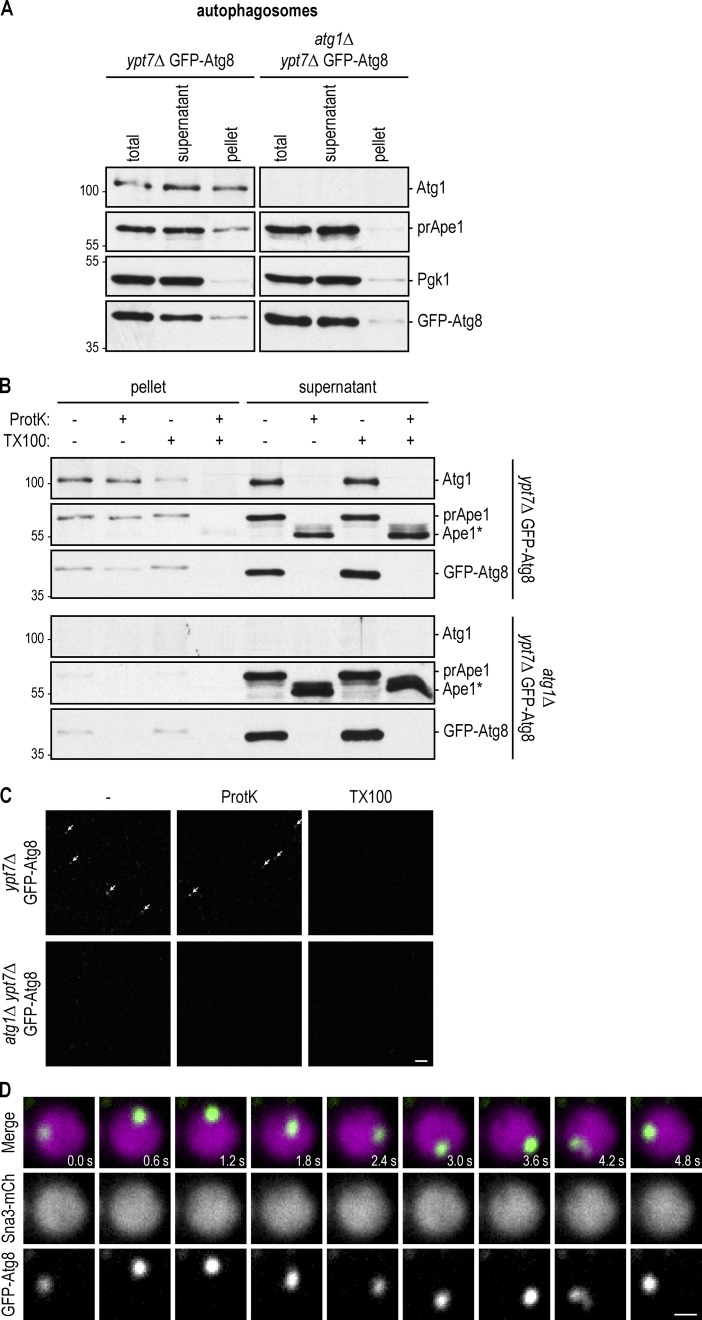
**Reconstitution of autophagosome–vacuole fusion in vitro. (A)** GFP-Atg8 *ypt7Δ* cells were starved for 16 h. After cell lysis, autophagosomes were enriched in a 20,000 *g* pellet and analyzed by anti-Atg1, anti-Ape1, anti-Pgk1, and anti-GFP Western blotting. *atg1Δypt7Δ* cells that cannot form autophagosomes served as a control. One representative experiment out of three is shown. **(B)** Samples from A were subjected to proteinase K (ProtK) and Triton X-100 (TX100) treatment as indicated and analyzed by anti-Atg1, anti-Ape1, and anti-GFP Western blotting. Ape1*, proteinase K–resistant fragment of Ape1. One representative experiment out of three is shown. Molecular masses are given in kilodaltons. **(C)** Samples from A were subjected to proteinase K and Triton X-100 treatment as indicated and analyzed by fluorescence microscopy. Arrows point to autophagosomes. One representative experiment out of three is shown. Bar, 10 µm. **(D)** The autophagosomal, vacuolar, and cytosolic fractions from Figs. 1 (C and D) and 2 A were coincubated together with an energy regeneration system for 2 h. Fusion was monitored by fluorescence microscopy and judged by the appearance of a mobile green dot in the vacuole. Shown are stills of a 12-s time-lapse video (Video 1). Bar, 0.5 µm.

To enrich for autophagosomes, we increased their abundance by inducing autophagy via starvation in cells lacking the Rab7 GTPase homologue Ypt7. *ypt7Δ* cells are deficient in vacuole–vacuole and autophagosome–vacuole fusion and thus accumulate mature autophagosomes (Kirisako et al., 1999). In addition, the autophagosomal marker GFP-Atg8 was expressed in these cells to allow autophagosome visualization in fluorescence microscopy. We removed the cell wall and lysed the cells as described above and enriched autophagosomes via 20,000 *g* centrifugation. We analyzed the fractions by Western blot analysis, monitoring the autophagosomal proteins GFP-Atg8, preApe1 (selective autophagy cargo), and Atg1 (Kraft et al., 2012) as well as the cytosolic marker Pgk1. The pellet displayed elevated levels of all three autophagosomal proteins relative to Pgk1, suggesting the successful enrichment of autophagosomes (Fig. 2 A). We did not find preApe1 enriched in the pellet of the control strain *atg1Δypt7Δ*, which lacks Atg1 and thus does not form autophagosomes (Fig. 2 A). However, we did detect enrichment of GFP-Atg8 in the *atg1Δypt7Δ* pellet (Fig. 2 A).

We used Western blotting to monitor the sensitivity of GFP-Atg8 and preApe1 to proteinase K treatment. GFP-Atg8 and preApe1 within sealed autophagosomes are protected from protease cleavage. However, if autophagosomes do not form or are incomplete, these proteins will be accessible to proteases. Atg8 will be degraded, whereas Ape1 will be processed to a 50-kD protease-resistant fragment. Indeed, GFP-Atg8 and preApe1 within the cytosolic fractions from *ypt7Δ* and *atg1Δypt7Δ* and GFP-Atg8 in the pellet isolated from *atg1Δypt7Δ* cells were sensitive to proteinase K treatment (Fig. 2 B). This is consistent with these fractions lacking mature autophagosomes and indicates that the GFP-Atg8 signal in the *atg1Δypt7Δ* pellet (Fig. 2 A) represents immature membranes with conjugated GFP-Atg8. In contrast, GFP-Atg8 and preApe1 in the autophagosome-enriched pellet isolated from *ypt7Δ* cells were largely protease resistant as expected for mature autophagosomes (Fig. 2 B).

Corroborating these findings, fluorescence microscopy analysis revealed green foci within the *ypt7Δ* pellet but not the *atg1Δypt7Δ* pellet, suggesting that only *ypt7Δ* cells accumulate GFP-Atg8–containing autophagosomes. These foci were resistant to proteinase K treatment but dissolved upon addition of the membrane-destabilizer Triton X-100, providing further support that foci represent mature and sealed autophagosomes containing GFP-Atg8 (Fig. 2 C).

Next, we determined whether these three fractions could reconstitute autophagosome–vacuole fusion in vitro. We isolated vacuoles from cells lacking the vacuolar lipase Atg15, which is required to degrade the autophagic body. Loss of Atg15 is expected to stabilize autophagic bodies within the vacuole and allow their visualization (Fig. 1 A). We incubated the autophagosome fraction from GFP-Atg8 *ypt7Δ* cells, the vacuole fraction from Sna3-mCherry *atg15Δ* cells, and the 20,000 *g* WT cytosolic fraction in the presence of ATP, phosphocreatine, and creatine kinase, which allow the generation of ATP from ADP, thus maintaining high ATP levels. We used fluorescence microscopy to quantify the fusion of GFP-Atg8–positive autophagosomes with Sna3-mCherry vacuoles. This precludes that other fusion events interfere with our analysis. We observed the appearance of highly mobile puncta representing GFP-Atg8–positive autophagic bodies (green) in the lumen of the vacuoles (Fig. 2 D, magenta, and Video 1). These findings establish that autophagosome–vacuole fusion can be reconstituted in vitro using a three-component system.

**Figure 3.**
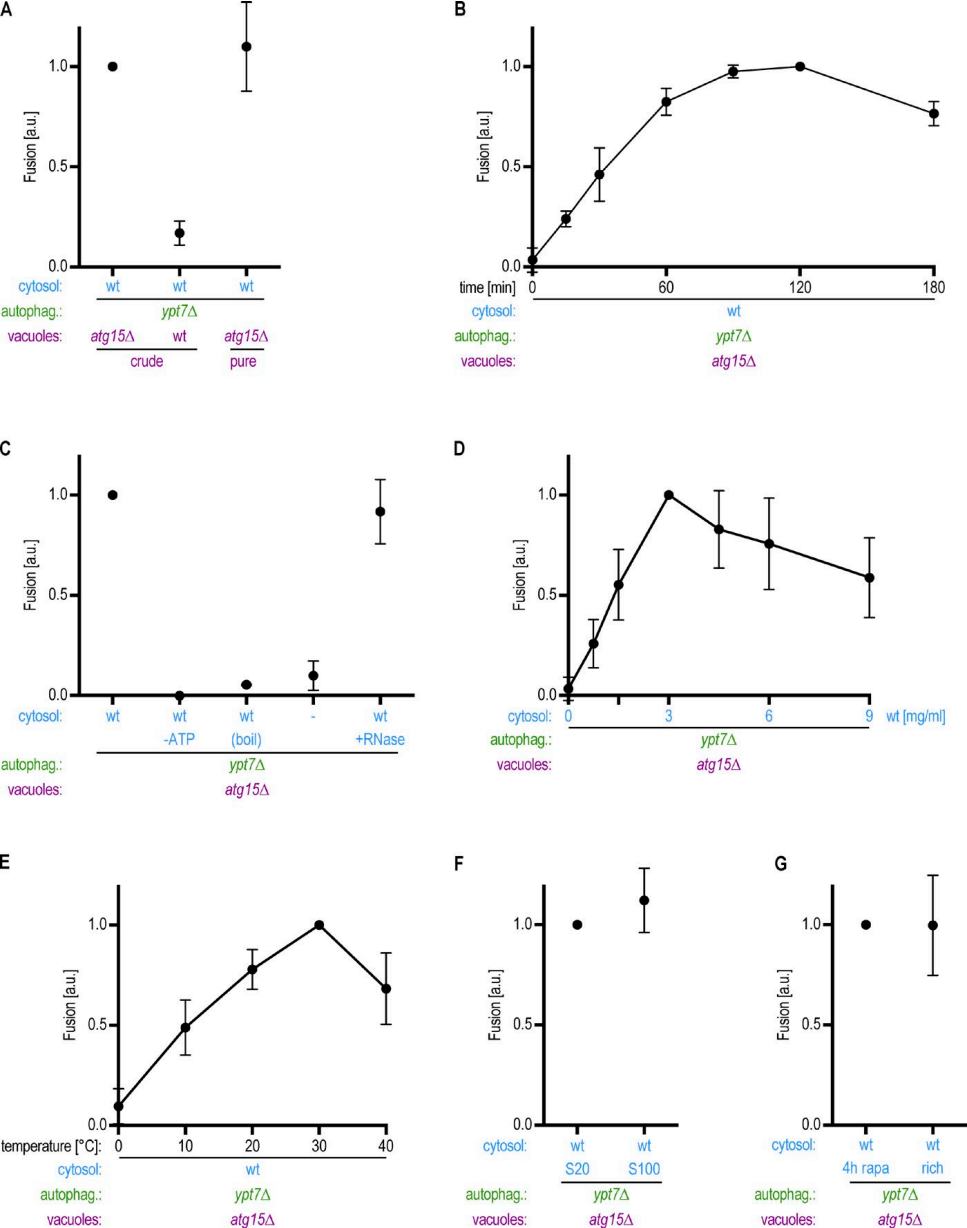
**Autophagosome–vacuole fusion depends on time, temperature, and cytosol concentration and requires energy. (A)** Vacuoles containing or lacking Atg15 as indicated were analyzed in an in vitro fusion assay as described in Fig. 2 D. **(B)** Samples as described in Fig. 2 D were incubated for the time indicated, and autophagosome–vacuole fusion was quantified. **(C)** Fusion reactions were performed in the presence and absence of an ATP regeneration system and with or without the prior incubation of the cytosolic fraction for 5 min at 95°C (boil) or RNase treatment. Dash indicates that no cytosol was added. **(D)** The amount of cytosol added to the fusion reaction was titrated as indicated. **(E)** Fusion reactions were performed at different temperatures as indicated. **(F)** Comparison of a 20,000 *g* and a 100,000 *g* cytosolic supernatant in the fusion reaction. **(G)** Comparison of cytosol prepared from logarithmically growing cells (rich) with cytosol from cells that had been treated with 220 nm rapamycin for 4 h before harvesting (rapa). All graphs show the mean from at least three independent experiments. Error bars are SD.

Using the same fluorescence microscopy approach, we quantified the efficiency of autophagosome–vacuole fusion. As expected, we did not detect GFP-Atg8–positive autophagic bodies within vacuoles containing Atg15, likely due to their rapid degradation in the vacuole (Fig. 3 A). We found that the fraction of *atg15Δ* vacuoles containing autophagic bodies increased for up to 2 h but then decreased, likely due to the limited stability of the vacuolar structures in vitro (Fig. 3 B; Cabrera and Ungermann, 2008). Efficient fusion required the cytosolic fraction and an ATP regeneration system (Fig. 3 C, Fig. S1 A, and Video 2). Fusion events furthermore depended on the concentration of cytosol (Fig. 3 D) and on the temperature (Fig. 3 E). Heating the cytosolic fraction to 95°C for 5 min (boil) before reconstitution abrogated fusion, suggesting that the cytosolic factor or factors supporting fusion is a heat-sensitive protein (Fig. 3 C). RNase treatment of the cytosol did not affect fusion efficiency. Fusion events were equally efficient with supernatants from 100,000 *g* high-speed and 20,000 *g* centrifugation serving as the cytosolic fraction, suggesting that the essential cytosolic factor is soluble (Fig. 3 F). Finally, the fusion frequency was similar with cytosolic fractions isolated from cells grown under nutrient-rich conditions and from rapamycin-treated cells (Fig. 3 G), suggesting that elevated autophagy is not required to induce the fusion signal. Taken together, we show that autophagosome–vacuole fusion can be recapitulated in vitro, with *ypt7Δ* autophagosomes, *atg15Δ* vacuoles, energy, and at least one soluble cytosolic factor.

### Phosphatidylinositol 3 (PI3)-phosphate (PI3P) is required for recruitment of the Rab GTPase Ypt7 to autophagosomes

**Figure 4.**
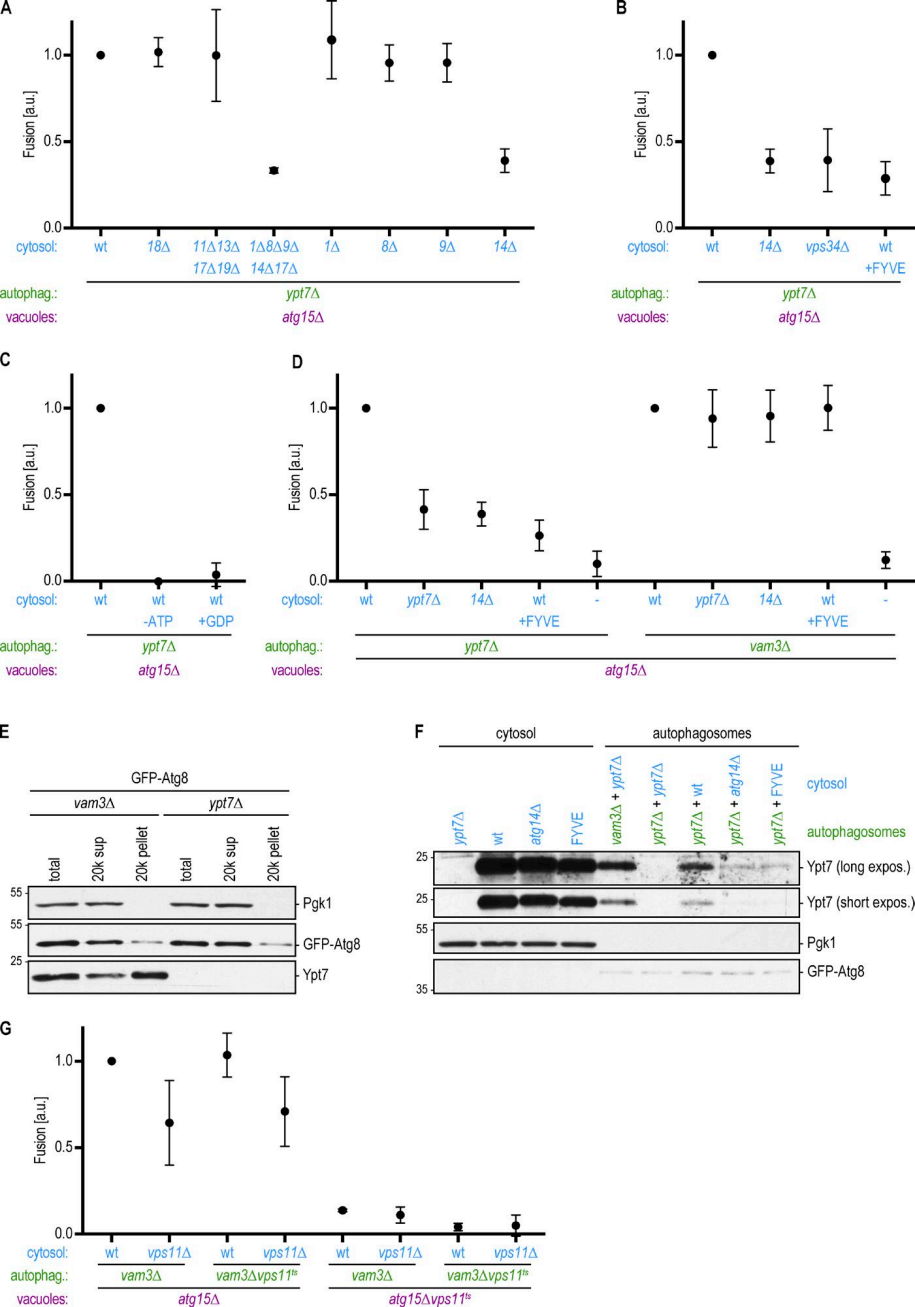
**The PI3-kinase complex I is required for the recruitment of Ypt7 to autophagosomes to promote autophagosome–vacuole fusion. (A)** Fusion assay as in Fig. 2 D. Cytosol was prepared from the indicated deletion strains. **(B)** Fusion assay as in Fig. 2 D. Cytosol was prepared from *atg14Δ* or *vps34Δ* cells or from cells overexpressing the FYVE domain. **(C)** Fusion assay as in Fig. 2 D. Fusion reactions were either mock treated or incubated without an ATP regeneration system or with 10 mM GDP. **(D)** Fusion assay as in Fig. 2 D. Cytosol and autophagosomes were prepared from the indicated deletion strains. The FYVE domain was overexpressed where indicated. **(E)** Autophagosomes were isolated from the indicated strains as in Fig. 2 A. The presence of Ypt7 in the autophagosome-enriched pellet was monitored by Western blotting. One representative experiment out of three is shown. **(F)** Autophagosomes were isolated from the indicated strains as in Fig. 2 A, incubated with cytosolic fractions as indicated, and again isolated by centrifugation. The binding of Ypt7 to the autophagosomes was assessed by Western blotting. One representative experiment out of three is shown. Molecular masses are given in kilodaltons. **(G)** Fusion assay as in Fig. 2 D. Cytosol, autophagosomes, and vacuoles were prepared from the indicated deletion or ts strains at 24°C. Fusion reactions were incubated at the restrictive temperature (30°C) for 2 h. All graphs show the mean from at least three independent experiments. Error bars are SD.

Next, we asked whether any of the Atg proteins within the cytosolic fraction are required for autophagosome–vacuole fusion in our system. We tested different Atg proteins from the main functional groups for their involvement in autophagosome fusion: Atg1, Atg13, and Atg17 (Atg1–ULK1 complex); Atg11 and Atg19 (Cvt pathway); Atg9 and Atg18 (Atg9 and Atg2-18 system); Atg14 (PI3-kinase complex I); and Atg8. To screen for the involvement of these proteins, we prepared cytosolic fractions from multiple knockout strains. We found that *atg11Δatg13Δatg17Δatg19Δ* and *atg18Δ* cytosolic fractions supported fusion, whereas the *atg1Δatg8Δatg9Δatg14Δatg17Δ* cytosolic fraction did not. These data suggest that Atg1, Atg8, Atg9, and/or Atg14 in the cytosolic fraction are necessary for autophagosome-to-vacuole fusion. Our analysis of the single knockout strains revealed that of these, only Atg14 was required in the cytosolic fraction to support fusion of *ypt7Δ* autophagosomes with *atg15Δ* vacuoles (Fig. 4 A).

Atg14 together with Vps34, Vps15, Atg6, and Atg38, forms the autophagy-specific PI3-kinase complex I (Kametaka et al., 1998; Araki et al., 2013), which phosphorylates position 3 of phosphatidylinositol to form PI3P (Schu et al., 1993). PI3P is essential for autophagy (Kihara et al., 2001), but it also functions in other pathways, for instance, by aiding in the stabilization of Rab proteins at their place of action (Simonsen et al., 1998). To test whether Atg14 is required for fusion due to its role in the PI3-kinase complex, we determined whether cytosolic fractions isolated from *vps34Δ* cells would support fusion. We found that fusion was strongly diminished with cytosolic fractions lacking Vps34 (Fig. 4 B). We postulate that residual Vps34 in the autophagosomal or vacuolar fractions might underlie the fusion events observed with the *vps34Δ* cytosolic fraction. To corroborate our findings, we isolated the cytosolic fraction from cells that overexpress the FYVE zinc finger domain, which competes for PI3P binding (Gillooly et al., 2000). Fusion was blocked with cytosolic fractions containing elevated levels of FYVE, further suggesting that PI3P activity supplied by the cytosolic fraction is required for autophagosome–vacuole fusion in this system (Fig. 4 B).

PI3P has been reported to stabilize Ypt7 on autophagosomes and at vacuoles (Hegedűs et al., 2016; Gao et al., 2018a). Ypt7, together with its guanine nucleotide exchange factor, the Mon1–Ccz1 complex, is required for autophagosome–vacuole fusion (Kim et al., 1999; Kirisako et al., 1999; Meiling-Wesse et al., 2002; Wang et al., 2002). Indeed, our isolation procedure capitalizes on the cytoplasmic accumulation of sealed autophagosomes in *ypt7Δ* cells (Kim et al., 1999). Autophagosomes lacking Ypt7 are fusion competent in our in vitro system, but Ypt7 is required for autophagosome–vacuole fusion in live cells. Together, these observations suggest that Ypt7 is supplied by the cytosol and/or the vacuole fraction to support the fusion of *ypt7Δ* autophagosomes in vitro.

To verify that GTPase activity is required for autophagosome–vacuole fusion in vitro, we added excess GDP to the fusion assay to lock GTPases in their inactive, GDP-bound state (Zick and Wickner, 2016; Langemeyer et al., 2018). Indeed, fusion was completely abolished in the presence of high GDP levels, consistent with a requirement for Ypt7 GTPase activity (Fig. 4 C). To evaluate whether Ypt7 within the cytosolic fraction is required for fusion in our system, we prepared cytosol from *ypt7Δ* cells. We discovered a 60% reduction in fusion between *ypt7Δ* autophagosomes and Ypt7-positive vacuoles upon loss of Ypt7 from the cytosol (Fig. 4 D). We speculate that the residual fusion is due to Ypt7 still present in the vacuolar fraction; however, we were unable to test this because loss of Ypt7 led to high fragmentation of vacuoles and impaired their quantification by fluorescence microscopy. Together, these data suggest that Ypt7 from the cytosolic fraction is recruited to *ypt7Δ* autophagosomes and that Ypt7 on both autophagosomes and vacuoles is required for fusion.

We hypothesized that cytosolic PI3P activity is required to stabilize Ypt7 recruitment to *ypt7Δ* autophagosomes in our system. To test this hypothesis, we determined whether autophagosomes prepared from a Ypt7-containing strain can bypass the requirement for PI3P. We isolated autophagosomes from fusion-deficient *vam3Δ* cells instead of from *ypt7Δ* cells. Vam3 is a SNARE protein required for autophagosome–vacuole fusion and is not predicted to influence Ypt7 localization. Similar to *ypt7Δ* cells, *vam3Δ* cells accumulate sealed autophagosomes in the cytoplasm (Darsow et al., 1997). We found that *vam3Δ* autophagosomes were proficient in fusing with *atg15Δ* vacuoles in our assay and that fusion depended on the cytosolic fraction (Fig. 4 D). However, unlike *ypt7Δ* autophagosomes, the *vam3Δ* autophagosomes also fused with vacuoles in cytosolic fractions that lack Ypt7 or Atg14 or that contain elevated levels of FYVE (Fig. 4 D). Western blotting of the autophagosomal fractions from *vam3Δ* and *ypt7Δ* cells confirmed that Ypt7 is recruited to *vam3Δ* autophagosomes (Fig. 4 E). Further, we found that Ypt7 was efficiently recruited to *ypt7Δ* autophagosomes incubated with a WT cytosolic fraction, but its recruitment was severely reduced in the absence of Atg14 or the presence of FYVE (Fig. 4 F). Collectively, these data suggest that Ypt7 is recruited to the autophagosome dependent on PI3P, likely via the action of Mon1–Ccz1 (Kinchen and Ravichandran, 2010; Nordmann et al., 2010).

Ypt7 in its activated, GTP-bound state binds to the HOPS tethering complex (Seals et al., 2000). The HOPS complex contains two Rab binding sites at opposite ends and recognizes selected SNAREs. It has therefore been suggested to bridge membranes (Reggiori and Ungermann, 2017). HOPS function has been reported to be required for autophagy (Scott et al., 1997; Chen et al., 2014). To determine whether and where the HOPS complex is required for autophagosome–vacuole fusion in vitro, we prepared the cytosolic fraction from HOPS mutant *vps11Δ* cells and autophagosomes and vacuoles from cells containing a temperature-sensitive (ts) variant of the HOPS component Vps11 (Rieder and Emr, 1997). We isolated autophagosomes and vacuoles at the permissive temperature but performed the fusion reaction at the restrictive temperature. Indeed, in the absence of HOPS function, fusion was decreased, supporting the involvement of the HOPS tethering complex during these late steps of autophagy (Figs. 4 G and S1 B). HOPS function was only required on vacuoles but not the autophagosome or cytosol. Reconstitution of HOPS function by recruitment of Vps11 from the cytosol does not seem to happen in our reconstitution assay, likely due to the use of the mutant Vps11ts protein, which occupies the binding space on the vacuole.

In summary, these findings suggest that the Atg14–Vps34 PI3-kinase complex is the only ATG module required for autophagosome–vacuole fusion. Our data suggest that the PI3-kinase complex generates PI3P on the autophagosome, which is required to recruit the Ypt7 system. The HOPS tethering complex is then required to promote fusion. Our data further suggest that other modules of the autophagy core machinery such as Atg8, the Atg1 kinase complex, and the Atg9 and Atg2–Atg18 systems are dispensable, at least in the cytosolic fraction, for fusion.

### The R-SNARE Ykt6 acts on the autophagosome to mediate autophagosome–vacuole fusion together with the Q-SNAREs Vam3, Vam7, and Vti1 on the vacuole

**Figure 5.**
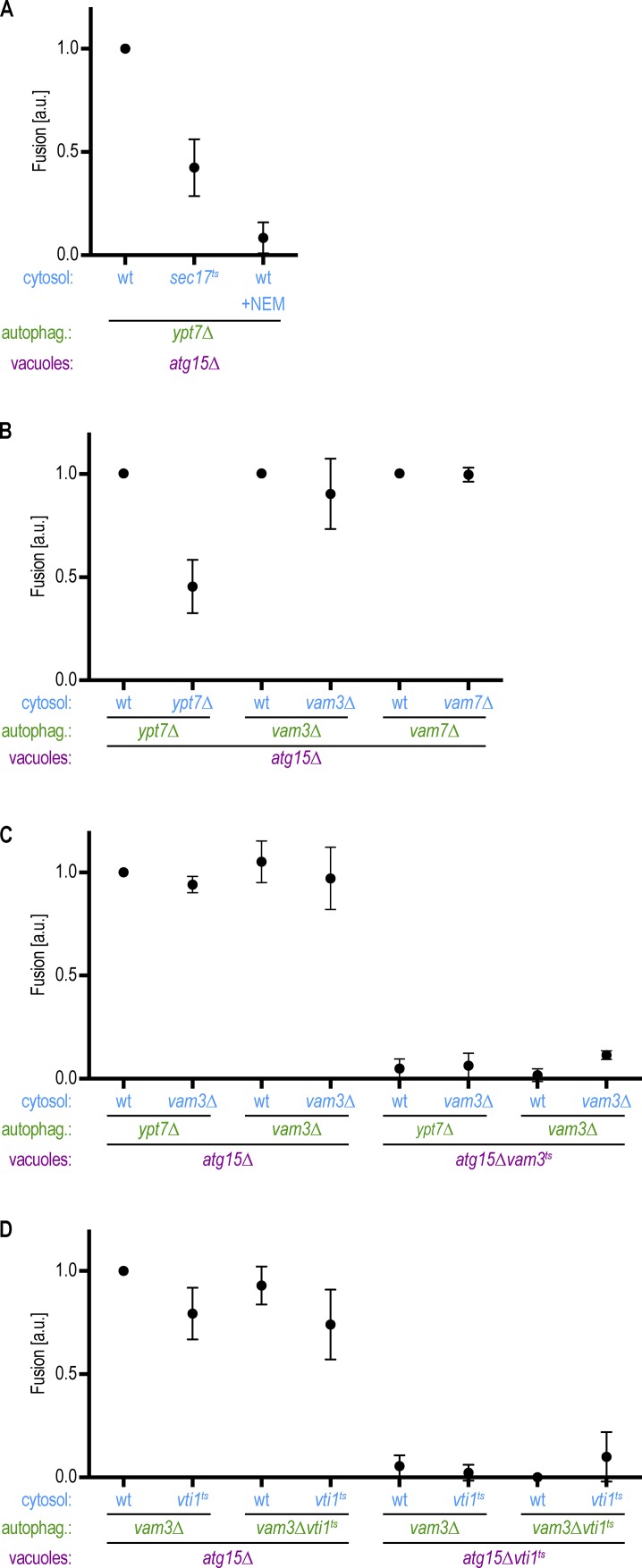
**The Q-SNAREs Vam3, Vam7, and Vti1 act on the vacuole to promote autophagosome–vacuole fusion. (A)** Fusion assay as in Fig. 2 D. Cytosol was prepared from the indicated deletions strains. The Sec18 inhibitor 5 mM N-ethylmaleimide (NEM) was added where indicated. **(B)** Fusion assay as in Fig. 2 D. Cytosol and autophagosomes were prepared from the indicated deletions strains. **(C and D)** Fusion assays as in Fig. 2 D. Cytosol, autophagosomes, and vacuoles were prepared from the indicated deletion or ts strains at 24°C. Fusion reactions were incubated at the restrictive temperature (30°C) for 2 h. All graphs show the mean from three independent experiments. Error bars are SD.

Mutations in the Q-SNAREs Vam3, Vam7, and Vti1, result in the accumulation of mature autophagosomes in the cytoplasm in vivo, suggesting that these factors are required for autophagosome–vacuole fusion (Darsow et al., 1997; Sato et al., 1998; Ishihara et al., 2001). SNARE-mediated fusion requires the actions of α-SNAP and N-ethylmaleimide–sensitive fusion protein (NSF), which in yeast are Sec17 and Sec18, respectively. We found that fusion between *ypt7Δ* autophagosomes and *atg15Δ* vacuoles was reduced upon incubation with a *sec17ts* cytosolic fraction at the nonpermissive temperature and in reactions containing the Sec18 inhibitor N-ethylmaleimide (Fig. 5 A; Glick and Rothman, 1987). These data provide further support that fusion in vitro and in vivo requires the action of SNARE proteins. However, which SNAREs act on the autophagosome or the vacuole and the nature of the R-SNARE within the complex remain elusive. Our in vitro reconstitution system allows us to dissect the functionally relevant subcellular localization of SNAREs within the fusion pathway and to uncover which R-SNARE is involved in the trans-SNARE complex.

First, we tested the requirement of Vam3 and Vam7 in the cytosolic fraction and on autophagosomes. Whereas incubation of *ypt7Δ* autophagosomes and *ypt7Δ* cytosol with *atg15Δ* vacuoles revealed diminished fusion, *vam3Δ* and *vam7Δ* autophagosomes fused efficiently with *atg15Δ* vacuoles in their respective cytosol (Fig. 5 B). These data suggest that unlike Ypt7, the SNAREs Vam3 and Vam7 are not required on autophagosomes and act on only the vacuolar side during fusion. To directly evaluate the requirement for Q-SNAREs on the vacuole, we used ts mutants because deletions led to strong fragmentation of vacuoles and impaired quantification by fluorescence microscopy. We isolated autophagosomes, vacuoles, and cytosol at the permissive temperature and performed the fusion reactions at the restrictive temperature. We detected fusion between *vam3Δ* autophagosomes and *atg15Δ* vacuoles, but not between *vam3Δ* autophagosomes and *atg15Δvam3ts* vacuoles, with both WT or *vam3Δ* cytosolic fractions. These data corroborate that fusion requires vacuolar localization of the Q-SNARE Vam3 (Fig. 5 C). Similar results were obtained with *vti1ts* mutant vacuoles (Fig. 5 D). Taken together, these findings suggest that autophagosome–vacuole fusion requires vacuolar localization and activity of the Q-SNAREs Vam3, Vam7, and Vti1.

Vesicle fusion requires the assembly of trans-SNARE complexes, which consist of three Q-SNAREs and one R-SNARE. The R-SNARE required for autophagosome–vacuole fusion, however, remains unidentified. Budding yeast contains five R-SNAREs: Sec22, Ykt6, Nyv1, Snc1, and Snc2. Snc1 and Snc2 are dispensable for bulk autophagy function (Nair et al., 2011), and Nyv1 has been reported to be dispensable for selective autophagy (Fischer von Mollard and Stevens, 1999). Both Sec22 and Ykt6 show a block in bulk autophagy (Nair et al., 2011). Vam3, Vam7, and Vti1 have been reported to act together with the R-SNARE Nyv1 during homotypic vacuole fusion (Ungermann et al., 1999), whereas Vti1 was found together with two other Q-SNAREs, Sft1 and Sed5, and the R-SNARE Ykt6 at the Golgi (Søgaard et al., 1994; Lupashin et al., 1997). Further, Vti1, Vam3, and Vam7 were detected with the R-SNAREs Nyv1 and Ykt6 on vacuoles (Ungermann et al., 1999). Based on these observations, we hypothesized that the R-SNAREs Sec22, Nyv1, and/or Ykt6 are required for autophagosome–vacuole fusion.

**Figure 6.**
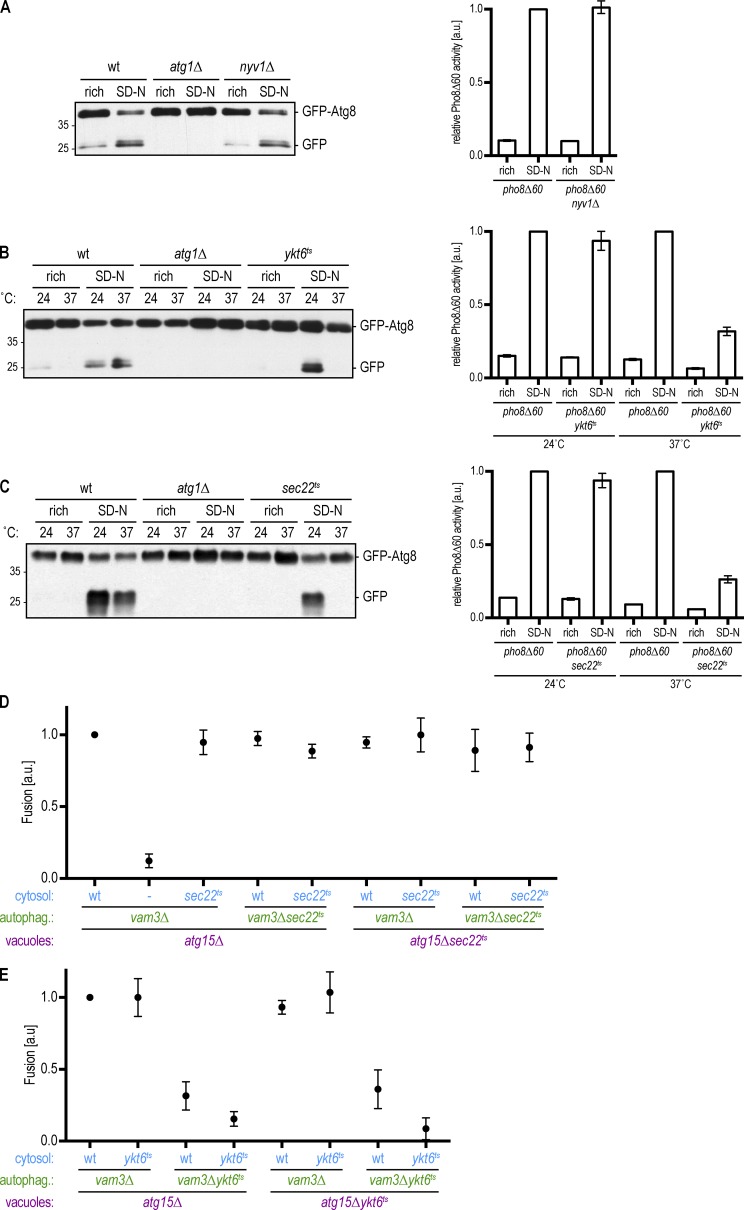
**The R-SNARE Ykt6 acts on the autophagosome to promote autophagosome–vacuole fusion. (A)** Left: The indicated strains expressing GFP-Atg8 were grown to logarithmic phase (rich) and were subsequently starved for 4 h (SD-N). TCA extracts were prepared, and GFP-Atg8 cleavage was monitored by anti-GFP Western blotting. One representative experiment out of three is shown. Right: Pho8Δ60 assay of *nyv1Δ* cells. Indicated cells were grown to mid–log phase and starved for 4 h where indicated. Pho8Δ60-specific alkaline phosphatase activity was measured in three independent experiments, and the mean was plotted normalized to starved *pho8Δ60* alkaline phosphatase activity. **(B and C)** Left: The indicated strains expressing GFP-Atg8 were grown at 24°C to logarithmic phase. Cultures were split and incubated for 4 h at the permissive (24°C) or restrictive (37°C) temperature, with (SD-N) or without starvation (rich) as indicated. TCA extracts were prepared, and GFP-Atg8 cleavage was monitored by anti-GFP Western blotting. One representative experiment out of three is shown. Right: Pho8Δ60 assay. The indicated strains were grown at 24°C to logarithmic phase. Cultures were split and incubated for 4 h at the permissive (24°C) or restrictive (37°C) temperature, with (SD-N) or without starvation (rich) as indicated. Pho8Δ60-specific alkaline phosphatase activity was measured in three independent experiments, and the mean was plotted normalized to starved *pho8Δ60* alkaline phosphatase activity at the respective temperature. Molecular masses are given in kilodaltons. **(D and E)** Fusion assay as in Fig. 2 D. Cytosol, autophagosomes, and vacuoles were prepared from the indicated deletion or ts strains at 24°C. Fusion reactions were incubated at the restrictive temperature (30°C) for 2 h. All graphs show the mean from at least three independent experiments. Error bars are SD.

Nyv1 is dispensable for the selective Cvt pathway (Fischer von Mollard and Stevens, 1999), but whether it is required for bulk autophagy is not known. We analyzed bulk autophagy in *nyv1Δ* cells first by monitoring the cleavage of GFP-Atg8 (Meiling-Wesse et al., 2002). We induced bulk autophagy by nitrogen starvation and observed cleavage of GFP-Atg8 in WT cells but not in *atg1Δ* cells as expected. GFP-Atg8 was cleaved in *nyv1Δ* cells upon induction of bulk autophagy (Fig. 6 A, left). In addition, we performed a Pho8Δ60 assay, which monitors the autophagic delivery of the Pho8Δ60 phosphatase into the vacuole (Noda et al., 1995). Both *nyv1Δ* and WT cells displayed Pho8Δ60 activity (Fig. 6 A, right). Thus, Nyv1 is dispensable for bulk autophagy function.

Ykt6 and Sec22 show high sequence similarity, and Ykt6 has been reported to be able to substitute Sec22 function during ER–Golgi transport (Liu and Barlowe, 2002). Furthermore, Ykt6 and Sec22 act together with the SNARE proteins Sso1 and Sec9 in Atg9 trafficking during early steps of autophagy (Nair et al., 2011). In agreement with these data, we found nitrogen-starved *ykt6ts* cells to be strongly impaired in GFP-Atg8 cleavage and in the delivery of Pho8Δ60 to the vacuole when grown at the restrictive temperature (37°C) but not at the permissive temperature (24°C; Fig. 6 B). We also observed impaired processing of Ape1, a readout for the selective Cvt pathway (Fig. S1 C; Harding et al., 1995). *sec22ts* mutant cells showed a defect in bulk autophagy similar to that of *ykt6ts* mutants (Fig. 6 C).

Ykt6 has also been speculated to act in later steps of autophagy such as autophagosome–vacuole fusion (Klionsky, 2005). However, because Ykt6 and Sec22 mutants trigger an early autophagy defect that abrogates autophagosome formation, their role in autophagosome–vacuole fusion cannot be determined from the analysis of intact cells. We therefore investigated whether Ykt6 and/or Sec22 are required for autophagosome–vacuole fusion in our in vitro reconstitution assay. We prepared autophagosomes from *vam3Δykt6ts* and *vam3Δsec22ts* cells grown at the permissive temperature. Control *vam3Δ* autophagosomes fused with *atg15Δ* vacuoles in the presence of WT, *sec22ts*, or *ykt6ts* cytosol, at the restrictive temperature. Additionally, *vam3Δ* autophagosomes fused with *atg15Δykt6ts* and *atg15Δsec22ts* vacuoles under these conditions. Whereas *vam3Δsec22ts* mutant autophagosomes fused normally with vacuoles at restrictive temperature, *vam3Δykt6ts* autophagosomes displayed substantially reduced fusion independent of the nature of the vacuolar and cytosolic fractions (Fig. 6, D and E). These findings strongly suggest that solely the R-SNARE Ykt6 is required on autophagosomes for fusion, whereas the R-SNARE Sec22 is dispensable during the autophagosome–vacuole fusion process. Taken together, our findings reveal that autophagosome–vacuole fusion requires the action of the Q-SNAREs Vam3, Vam7, and Vti1 on the vacuole and the R-SNARE Ykt6 on the autophagosome.

## Discussion

**Figure 7.**
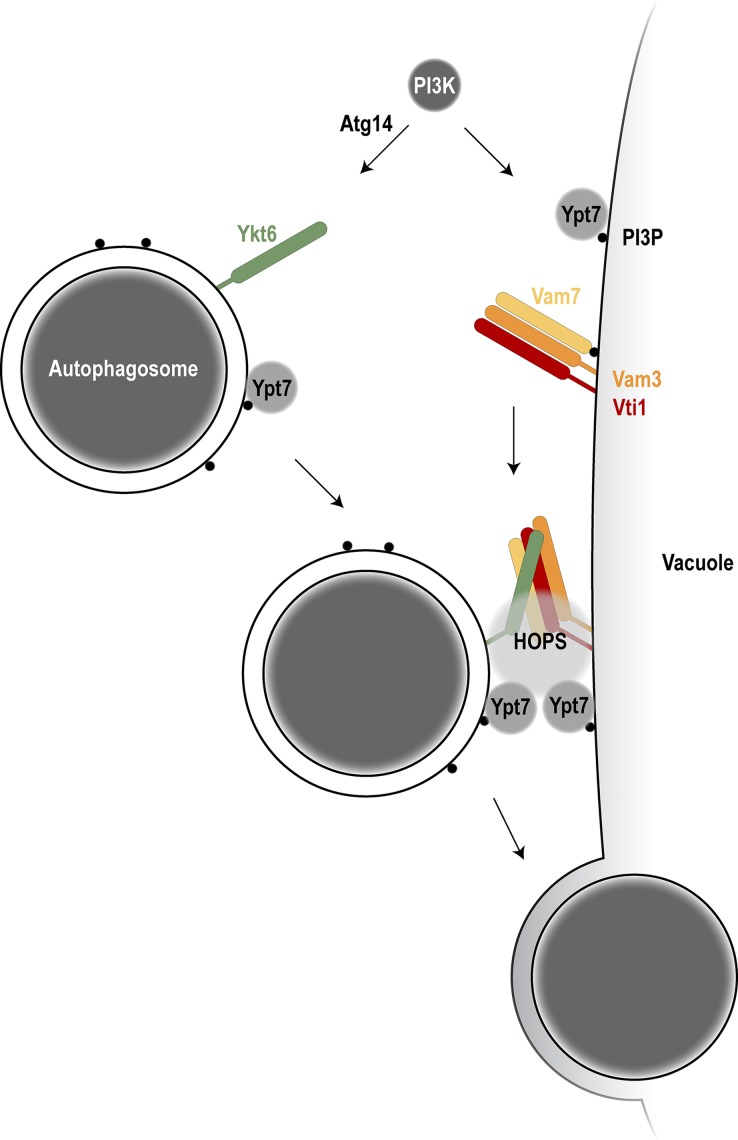
**Model of autophagosome–vacuole fusion.** Atg14 in the PI3-kinase complex I is required to produce PI3P on the autophagosome to allow Ypt7 recruitment. The R-SNARE Ykt6 localizes to autophagosomes; the Q-SNAREs Vam3, Vam7, and Vti1 localize to the vacuole. With the help of Ypt7 on both the autophagosome and the vacuole and the HOPS tethering complex, trans-SNARE complex formation is promoted and allows autophagosome–vacuole fusion.

We established an in vitro reconstitution system for autophagosome–vacuole fusion. This novel approach allowed us to identify the molecular players required for fusion as well as their place of action. Our system requires three components to promote fusion in vitro: crude autophagosomes, vacuoles, and a cytosolic extract complemented with ATP. We found that the cytosol contributes Atg14 to the reaction, which acts within the Vps34 complex to generate PI3P and recruit the Ypt7 module to the autophagosome. The HOPS tethering complex recruited by Ypt7 is required to tether organelles and prepare SNARE proteins for fusion. We observed that also autophagosome–vacuole fusion depends on HOPS activity. We discovered that fusion requires the R-SNARE Ykt6 on the autophagosome, together with the Q-SNAREs Vam3, Vam7, and Vti1 on the vacuole (Fig. 7). These findings shed new light on the mechanism of autophagosome–vacuole fusion and revealed the yet-unknown role of the R-SNARE Ykt6 in this process. Work by the Ungermann laboratory reveals a similar mechanism of autophagosome–vacuole fusion, which further supports our findings (see Gao et al., 2018b, in this issue).

Our findings reveal that autophagosome–vacuole fusion uses a mechanistically related process to other fusion events such as vacuole–vacuole or endosome–vacuole fusion because it requires the action of Rab GTPases, SNARE proteins, and the HOPS tethering complex. It uses, however, a different set of SNARE proteins. SNARE proteins on early endosomes are Vti1, Tlg1/Tlg2, and Snc1/Snc2, and SNARE proteins on late endosomes are Pep12, Syn8, Vti1, and Ykt6. Therefore, autophagosome–vacuole fusion uses a related but distinct fusion process.

Atg14 acts as part of the PI3-kinase complex I together with Vps34 in generating PI3P. In this study, we find that Atg14 and Vps34 are required for autophagosome–vacuole fusion because together, they mediate the recruitment of the Mon1–Ccz1–Ypt7 complex to autophagosomes. Ypt7 is also required on the vacuole, where its recruitment also depends on PI3P. If vacuoles lack Ypt7 binding, they fragment. Because *atg14Δ* cells show normal, nonfragmented vacuoles, it is unlikely that Atg14 also mediates PI3P production on vacuoles. Recent work suggests that mammalian Atg14 interacts with STX17 and SNAP29 on mature autophagosomes and may facilitate efficient SNARE assembly during autophagosome–lysosome fusion (Diao et al., 2015).

In contrast with classical SNARE proteins, Vam7 lacks a transmembrane domain but binds to PI3P via a PX domain (Cheever et al., 2001). Vam7 has been reported to interact with the Atg17–Atg31–Atg29 complex and mediate autophagosome–vacuole fusion (Liu and Klionsky, 2016). However, whether Vam7 acts on the autophagosome or on the vacuole during this process was unclear. In this study, we report that Vam7, together with Vam3 and Vti1, is required to act on the vacuolar side in this fusion process. The interaction of Vam7 with the Atg17–Atg31–Atg29 complex already during early autophagy at the preautophagosomal structure (PAS) might prevent premature fusion of Vam7-containing membranes. How this potential block could be released upon completion of autophagosomes remains to be shown.

Ykt6 has been described to act in multiple intracellular fusion reactions. It acts during homotypic vacuolar fusion (Ungermann et al., 1999), in retrograde trafficking to the cis-Golgi (Søgaard et al., 1994), and in the alkaline phosphatase and the carboxypeptidase Y pathway (McNew et al., 1997). Ykt6 has also been reported to function early in autophagy as autophagosomes fail to form in Ykt6 mutants due to a failure in Atg9 vesicle trafficking (Nair et al., 2011). Ykt6 forms a complex with the SNAREs Vam3 and Vam7 in homotypic vacuole fusion (Ungermann et al., 1999) but with the SNAREs Sso1 and Sec9 during early steps of autophagy (Nair et al., 2011). In this study, we show that Ykt6 on autophagosomes acts during autophagosome–vacuole fusion, suggesting that this SNARE protein is required for multiple steps in autophagy.

In higher eukaryotes, the SNAREs Syntaxin 17, SNAP29, and VAMP8 are required for autophagosome–lysosome fusion, and Syntaxin 17 has been proposed to act on the autophagosome in the fusion process (Itakura et al., 2012). During the preparation of this manuscript, both the *D. melanogaster* and the mammalian Ykt6 homologues have been shown to also act on autophagosomes (Matsui et al., 2018; Takáts et al., 2018), suggesting that the function of Ykt6 on autophagosomes is evolutionarily conserved.

## Materials and methods

### Yeast strains and growth conditions

Yeast strains are listed in Table S1 (Li et al., 2011; Torggler et al., 2016). Mutations were integrated by homologous recombination into the deletion strain; genomic insertions (tagging) were performed according to Janke et al. (2004) or Longtine et al. (1998); and multiple deletions or mutations were generated by PCR knockout, mating, and dissection. GFP-ATG8–containing strains were generated by crossing to yTB281 (Torggler et al., 2016) or strains derived thereof, which had been generated by seamless tagging (Khmelinskii et al., 2011) in BY4741 and used to cross further strains.

Yeast cells were grown to mid–log phase as described (Papinski et al., 2014). Bulk autophagy was induced by nitrogen starvation in SD-N (0.17% yeast nitrogen base without amino acids and 2% glucose) or by treatment with 220 nM rapamycin.

### Cytosol preparation

Yeast cell extracts were prepared from cells treated with 220 nM rapamycin for 4 h by freezer milling as described (Torggler et al., 2016). The extracts were thawed on ice and cleared by spinning at 20,000 *g* for 30 min at 4°C. For preparing S100 cytosol, these cleared extracts were spun at 100,000 *g* for 30 min at 4°C, and the supernatant was used.

### Preparation of an autophagosome-enriched fraction

Yeast cells were grown in YPD to mid–log phase, followed by starvation for 16 h in SD-N. The cells were then harvested and incubated with 50 OD/ml DTT buffer (100 mM Tris-HCl, pH 9.4, and 10 mM DTT) for 15 min at 30°C, spun at 3,000 *g* for 5 min, resuspended with 50 OD/ml SP buffer (50 mM potassium phosphate, pH 7.5, 1 M sorbitol, and 0.25× YPD) and spheroplasted by lyticase (90 U/ml) treatment at 30°C for 30 min. Spheroplast formation was monitored by diluting the spheroplasts 1:100 in SP buffer or in H_2_O. Efficient spheroplasting causes a 10-fold drop at OD_600_ in the H_2_O sample. Spheroplasts were collected by centrifugation at 1,500 *g* for 10 min at 4°C, resuspended to 20 OD/ml in PS200 buffer (10 mM Pipes-KOH, pH 6.8, 200 mM sorbitol, and c0mplete protease inhibitor cocktail [Roche]), and lysed on ice by passing through a 22G needle 30 times. The lysate was cleared from unbroken cells by 500 *g* centrifugations at 4°C until no pellet was observed, and the cleared lysate was then spun at 20,000 *g* for 30 min at 4°C. The resulting pellet fraction was washed with PS200 buffer and resuspended to 10,000 OD/ml in PS200 buffer and used as autophagosome-enriched fraction.

### Preparation of vacuoles

Yeast cells were grown in YPD to mid–log phase, harvested, and resuspended to 50 OD/ml in DTT buffer. After incubation at 30°C for 15 min, they were spun at 3,000 *g* for 5 min, resuspended to 50 OD/ml in SP buffer, and spheroplasted by lyticase (60 U/ml) treatment at 30°C for 30 min. Spheroplast formation was monitored by a drop in OD_600_ as described above. Spheroplasts were then pelleted at 1,500 *g* for 10 min at 4°C, resuspended to 20 OD/ml in PS200 buffer, and lysed on ice by passing through a 22G needle 30 times. The lysate was cleared by 500 *g* centrifugations at 4°C until no pellet was observed, and the cleared lysate was then spun at 6,000 *g* for 30 min at 4°C. The resulting pellet fraction was washed with PS200 Buffer and resuspended to 2,500 OD/ml in PS200 buffer and used as crude vacuole fraction.

To obtain a pure vacuole fraction, crude vacuoles were prepared as described above from 1,000 OD cells, and the 6,000 *g* pellet was resuspended in 3 ml 15% Ficoll buffer (15% Ficoll, 10 mM Pipes-KOH, pH 6.8, and 200 mM sorbitol). The resuspended crude vacuoles were transferred into a SW40 centrifugation tube (Beckman Coulter) and overlaid with 3 ml of 8% Ficoll, 3 ml of 4% Ficoll, and 1 ml 0% Ficoll (all in 10 mM Pipes-KOH, pH 6.8, and 200 mM sorbitol). The step gradient was spun at 180,000 *g* at 4°C for 90 min, and the vacuoles were collected from the 0–4% interphase.

### In vitro fusion assay

Standard fusion reactions (20 µl) contained 25 OD equivalents of crude vacuoles, 50 OD equivalents of the autophagosome-enriched fraction, 3 mg/ml cytosol, and an ATP regeneration system (1 mM ATP, 0.1 mM GTP, 20 mM phosphocreatine, 0.5 mg/ml creatine kinase, and 1 mM Mg(OAc)_2_) in reaction buffer (20 mM Pipes-KOH, pH 6.8, 200 mM sorbitol, and 75 mM KOAc). After 120 min at 30°C the reactions were transferred to ice, and 10 µl of the reaction was mixed with 10 µl of 1% low melting agarose dissolved in reaction buffer (preheated to 45°C), immediately transferred onto glass slides, and covered with a coverslip. The slides were incubated at RT for 5 min for the agarose to solidify before visualization.

### Protease protection

Protease protection experiments were performed as previously described (Kraft et al., 2012; Papinski et al., 2014). In brief, the pellet and the supernatant fractions obtained from preparation of autophagosome-enriched fractions as described above were treated with or without proteinase K (50 µg/ml; AppliChem) and 0.2% Triton X-100 on ice. After 30 min of treatment, the samples were subjected to TCA precipitation and analyzed by Western blotting.

### Pho8Δ60 assay

15–25 OD units of yeast culture were harvested by centrifugation (2,000 *g* for 5 min). Pellets were washed in 1 ml distilled H_2_O, followed by centrifugation at 4°C and resuspension of the pellet in 2 ml ice-cold 0.85% NaCl containing 1 mM PMSF. After another centrifugation step, pellets were resuspended in 16 µl/OD unit lysis buffer (20 mM Pipes, pH 6.8, 0.5% Triton X-100, 50 mM KCl, 100 mM potassium acetate, 10 mM MgSO_4_, 10 µM ZnSO_4_, 1 mM PMSF, and c0mplete protease inhibitor cocktail [Roche]). Cells were lysed by bead beating, and extracts were cleared by centrifugation at 16,000 *g* for 5 min at 4°C. Protein concentration of the supernatant was adjusted to 50 µg in 100 µl lysis buffer. Samples were prepared in duplicates for enzymatic reactions and control reactions. 400 µl reaction buffer (0.4% Triton X-100, 10 mM MgSO_4_, 10 µM ZnSO_4_, and 250 mM Tris-HCl, pH 8.5) containing 6.25 mM α-naphthylphosphate (Noda and Ohsumi, 1998; Sigma-Aldrich) was added to enzymatic reactions, or only reaction buffer was added to control reactions. Reactions were incubated at 37°C for 10 min and stopped by adding 500 µl stop buffer (1 M glycine, pH 11). Fluorescence was measured using 345 nm for excitation and 472 nm for emission. The values of the control samples were subtracted from the values of the enzymatic reactions.

### Quantitative live-cell imaging

Fluorescent images were recorded with an applied precision PersonalDV microscope (Applied Precision) with an UPlanSApo 100×/1.4 oil objective, a CoolSNAP HQ2 camera, and an InsightSSI solid state illumination unit as time-lapse images of 0.6-s intervals with SoftWoRx software (Applied Precision; Figs. 2 D and S1 A) or with a AxioObserver Z1 inverted microscope (ZEISS) equipped with an α–Plan Apochromat 100×/1.46 oil differential interference contrast RMS objective, a PCO 1600 camera, and a Lumencor SOLA 6-LCR-SB light source with VisiView software (Visitron Systems), at RT. In Fig. 1 B, single slices from stacks of 0.4-µm-thick slices are shown after background subtraction with a rolling ball of 50-pixel radius. For the analysis of fusion events, the images were recorded as time lapses with 0.5-s intervals. Background subtraction was performed with a rolling ball of 50-pixel radius for the red channel (vacuoles) and with a 7-pixel radius for the green channel (autophagosomes). A representative example of such a time-lapse is shown in Figs. 2 D and S1 A as well as Videos 1 and 2. The number of red vacuoles with moving green puncta (GFP-Atg8–positive autophagic bodies) were counted blindly after randomizing the sample names. The total number of vacuoles was determined with software assistance: after background subtraction (as described above), an autothreshold based on Renyi’s entropy was applied, and particles above the threshold having a diameter >0.25 µm were counted as vacuoles. All image analyses were done using the ImageJ software (National Institutes of Health).

### Antibodies

The following antibodies were used in this study: mouse monoclonal anti-GFP antibody (Roche), mouse monoclonal anti-Pgk1 antibody (Invitrogen), mouse monoclonal anti-Vph1 (Abcam), mouse monoclonal anti-Pho8 (Abcam), and polyclonal rabbit anti-Ape1 antibody (Torggler et al., 2016). Polyclonal anti-Atg8 antibodies were generated by immunizing rabbits with recombinant GST-Atg8. Rabbit anti-Vti1 and anti-Ypt7 antibodies were a kind gift from C. Ungermann (University of Osnabrueck, Osnabrueck, Germany). The rabbit anti-Atg1 antibody was kindly provided by D. Klionsky (University of Michigan, Ann Arbor, MI), and the rabbit anti-Tom70 antibody was a kind gift by C. Meisinger (University of Freiburg, Freiburg, Germany).

### Plasmid construction

Plasmids are listed in Table S2 (Sikorski and Hieter, 1989; Kraft et al., 2008). pLB42 was constructed by PCR amplification of TagBFP from pDP103 (Pfaffenwimmer et al., 2014) and the 2×FYVE domain from pEGFP-2×FYVE (provided by H. Stenmark; Gillooly et al., 2000). These fragments were inserted into the EcoRI and SalI digested pRS316-Gal1 vector via Gibson annealing. An HA tag was added with the oligonucleotides used for amplification. pLB49 was constructed by subcloning the *vam3ts* mutant (provided by F. Reggiori, University Medical Center Groningen, Groningen, Netherlands) via BamHI-SalI into pRS315. pLB52 was generated by subcloning *vps11-1ts* into pRS315 from a plasmid obtained from W. Wickner (Geisel School of Medicine at Dartmouth, Hanover, NH).

### Statistical analysis

P values were calculated using the two-tailed Welch’s *t* test, apart from Fig. S1 C, in which the two-tailed Student’s *t* test was used. P values are listed in Table S3.

### Online supplemental material

Fig. S1 shows the fusion assay and selective autophagy experiments. Video 1 shows a time lapse of an autophagosome fused with a vacuole, and Video 2 shows a time lapse of a fusion reaction without cytosol. Table S1 describes the yeast strains, Table S2 describes the plasmids used in this study, and Table S3 lists the P values.

## Supplementary Material

Supplemental Materials (PDF)

Tables S1-S3 (ZIP)

Video 1

Video 2
