# Supplementary material for: Reconstitution reveals Ykt6 as the autophagosomal SNARE in autophagosome–vacuole fusion
Source: J Cell Biol. 2018 Oct 1;217(10):3656–69. doi: 10.1083/jcb.201804028 (PMC6168255; doi:10.1083/jcb.201804028)
Supplement: Supplemental Materials (PDF) [file JCB_201804028_sm.pdf]

Supplemental material

Bas et al., https://doi.org/10.1083/jcb.201804028

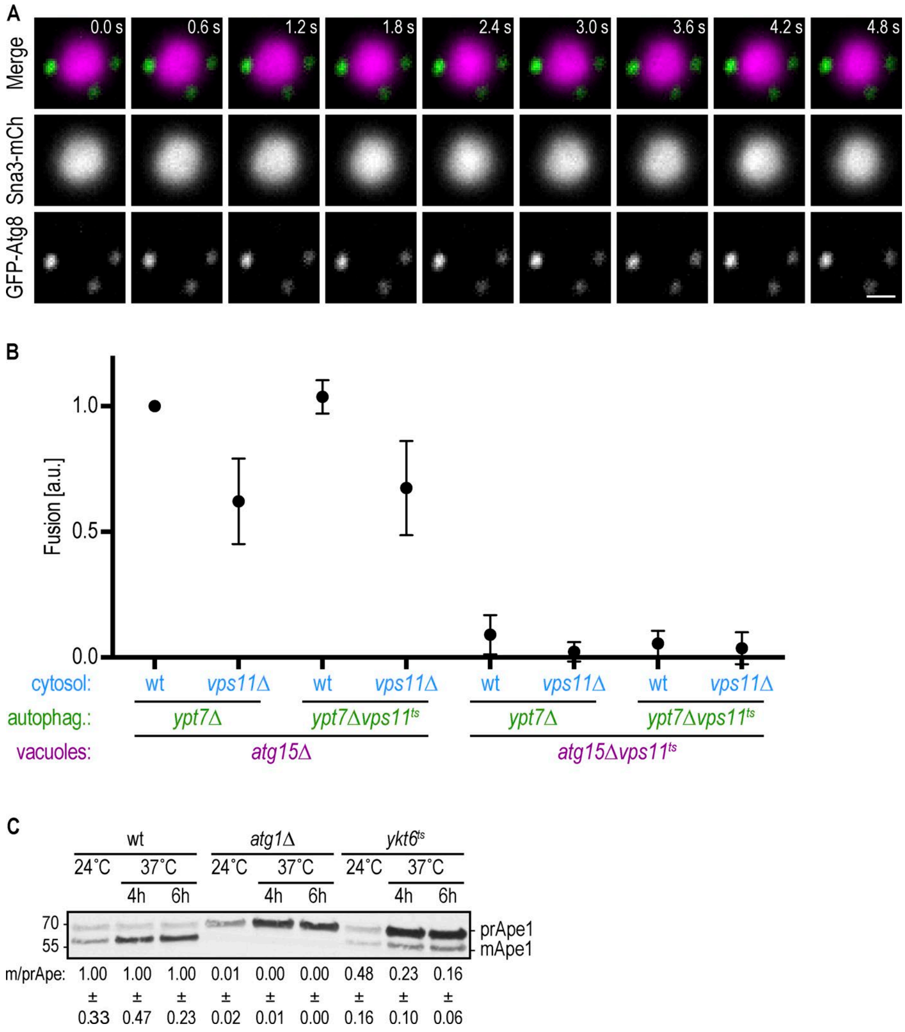

Figure S1. **Fusion and selective autophagy experiments. (A)** The fusion assay was performed as in Fig. 2 D, however, without the addition of cytosol. Shown are stills of a 12-s time-lapse video (Video 2). Bar, 0.5 µm. **(B)** Fusion assay as in Fig. 2 D. Cytosol, autophagosomes, and vacuoles were prepared from the indicated deletion or ts strains at 24°C. Fusion reactions were incubated at the restrictive temperature (30°C) for 2 h. The mean from three independent experiments is shown. Error bars are SD. **(C)** The indicated strains were grown at 24°C to logarithmic phase. Cultures were then incubated at the permissive (24°C) temperature or 4 or 6 h at the restrictive (37°C) temperature as indicated. TCA extracts were prepared, and Ape1 cleavage, which is a readout for a functional Cvt pathway, was monitored by anti-Ape1 Western blotting. One representative experiment out of three is shown as well as the mean mApe1/preApe1 ratio ± SD from all three experiments normalized to the respective WT sample. Molecular masses are given in kilodaltons.

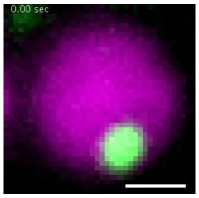

Video 1. **Time lapse of an autophagosome fused with a vacuole.** The autophagosomal, vacuolar, and cytosolic fractions from Figs. 1, C and D, and 2 A were coincubated together with an energy regeneration system for 2 h. Fusion was monitored by fluorescence microscopy and judged by the appearance of a mobile green dot in the vacuole. A time-lapse video was recorded for 12 s with 21 frames at 0.6-s intervals. The video is shown at a frame rate of seven frames per second. Visible is an autophagic body (GFP-Atg8; green) inside a yeast vacuole (Sna3-mCherry; magenta). Stills of the video are shown in Fig. 2 D. Bar, 0.5 µm.

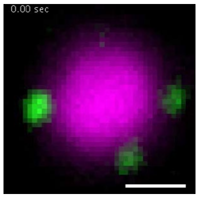

Video 2. **Time lapse of a fusion reaction without cytosol.** The fusion assay was performed as in Video 1 but without the addition of cytosol. Stills of the video are shown in Fig. S1 A. Bar, 0.5 µm.

Table S1 is a separate Excel document showing yeast strains used in this study.

Table S2 is a separate Excel document showing plasmids used in this study.

Table S3 is a separate Excel document showing P values from this study.
